# Skin Closure After No-Touch Saphenous Vein Harvest: Strategies to Minimize Wound Complications

**DOI:** 10.21470/1678-9741-2022-0143

**Published:** 2022

**Authors:** Min-Seok Kim, Seong Wook Hwang, Ki-Bong Kim

**Affiliations:** 1 Cardiovascular Center, Myongji Hospital, Goyang-si, Gyeonggi-do, Republic of Korea

**Keywords:** Coronary Artery Bypass, Venous Grafts, Tissue and Organ Harvesting, Saphenous Vein, Health Strategies.

## Abstract

The no-touch saphenous vein with surrounding pedicle tissue harvesting technique preserved endothelium and vessel wall integrity and demonstrated improved long-term saphenous vein conduit patency that was comparable to internal thoracic artery conduit patency. Despite improved saphenous vein conduit patency rates, there is a possibility that no-touch saphenous vein harvest may increase wound complication rates by increased tissue disruption, including venous and lymphatic channels. Comprehensive strategies to minimize leg wound complications after no-touch saphenous vein harvest are discussed.

**Table:** 

Abbreviations, Acronyms & Symbols	
ABI	= Ankle brachial index
CABG	= Coronary artery bypass grafting
CT	= Computed tomography
SV	= Saphenous vein

## INTRODUCTION

The saphenous vein (SV) conduit has been and still is widely used for coronary artery bypass grafting (CABG); however, its use as an aortocoronary bypass graft has shown declining patency with time, resulting in poorer clinical outcomes^[^1^]^. The no-touch SV harvesting technique, in which manipulation of the SV was minimized during harvesting and endothelium and vessel wall integrity were preserved, improved long-term SV conduit patency demonstrated to be comparable to internal thoracic artery conduit patency^[^2^,^3^]^. Despite improved SV conduit patency rates, it is possible that no-touch SV harvest may cause greater leg swelling and increased wound complications by disruption of more tissues, including venous and lymphatic channels^[^4^]^. Leg wound complication rates have been reported to be 6% to 23% in the no-touch SV groups, which were higher than in the conventionally harvested SV groups^[^4^-^6^]^. Strategies to minimize leg wound complications after no-touch SV harvest based on our experience are discussed below.

## DISCUSSION

### Preoperative and Intraoperative Evaluation Before Skin Incision

Preoperative thoracoabdominal multidetector computed tomography (CT) angiography was performed in patients undergoing CABG to assess their vascular status from the neck to femoral vessels due to their high atherosclerotic steno-occlusive risk^[^7^]^. If the patient had any exertional leg symptoms or had significant steno-occlusive disease of a lower extremity, an ankle brachial index (ABI) test was performed^[^8^]^. An ABI ratio of ≤ 0.7 in any lower limb suggested moderate to severe peripheral arterial disease and harvesting the ipsilateral SV was avoided to prevent skin wound complications. Lower extremity vein CT angiography was performed at the same time as thoracoabdominal CT without additional contrast material. Lower extremity vein CT angiography allowed thorough evaluation of lower extremity veins, such as of their course, size, and quality.

After anesthetic induction, Doppler ultrasonography mapping was performed to reassess the course, size, quality (such as ectatic change, sclerosis, or thrombosis), and tributaries of the SV^[^9^]^.

The SV from a lower leg was commonly chosen as opposed to the upper leg SV to decrease the possibility of size mismatch with native coronary arteries or internal thoracic arteries. SV with diameters between 2.5 mm and 4.5 mm and fewer tributaries were preferred for use.

### Skin Incision and No-Touch SV Harvest

The no-touch SV harvesting technique has been previously described^[^10^,^11^]^. Two or three interrupted open skin incisions were made along the SV course marked by surgical pens to avoid unnecessary and large dissections of the leg. One- to 2-cm intervening bridges of skin were left intact to allow better closure of the wound and to minimize ischemic changes along the skin edges. The no-touch SV harvest included surrounding pedicle tissue, whereby the SV pedicle was harvested along with an approximately 3- to 5-mm wide margin of adjacent adipose tissues on both sides of the SV and thin layers of adherent connective tissues posteriorly, in addition to minimized manipulation of the SV and avoidance of manual intraluminal dilatation. Side branches were divided after clipping. Titanium clips were applied at least 1 mm from branch origins to prevent inadvertent endothelial injury.

The no-touch SV harvest was performed using either an electrocautery, Harmonic Scalpel™ (Ethicon Endo-Surgery, Inc., Cincinnati, Ohio, United States of America), or LigaSure™ (Medtronic, Inc., Minneapolis, Minnesota, United States of America) device. During the no-touch SV harvesting using an electrocautery, the cautery setting was kept on low throughout the dissection to avoid thermal injury to the SV pedicle. No-touch SV harvest using either the Harmonic Scalpel™ or the LigaSure™ devices had advantages of better hemostasis and minimal thermal injury over the no-touch SV harvest using an electrocautery. The Harmonic Scalpel™ device uses ultrasonic vibration technology, and the LigaSure™ device is an electrothermal bipolar-activated device. Both devices provide effective hemostasis and minimal lateral thermal spread^[^12^]^.

### Wound Closure

After protamine administration to normalize the prolonged activated clotting time at the end of CABG, a Jackson-Pratt drain was inserted into the SV harvesting site and the leg wounds were closed in layers: interrupted 3-0 absorbable sutures to the subcutaneous tissue, interrupted 4-0 subcuticular absorbable sutures, and additional staples or zip surgical closure method for the skin. Zip skin closure (ZipLine Medical, Inc., Campbell, California, United States of America) is a noninvasive skin closure system, and it has several advantages for wound healing such as providing better cosmetic results, facilitating wound healing, and reducing infection risk^[^13^,^14^]^. An elasticated bandage was applied to the leg wound after surgery and was replaced by a compression stocking from the 2^nd^ postoperative day. The compression stocking was kept in place for 1 to 2 months postoperatively for prevention of leg swelling. The Jackson-Pratt drain was removed when the amount of drained fluid decreased to < 10 mL daily. Leg wound complications in the SV harvesting site were defined as infection or wound disruption that needed additional repair or secondary intention healing. Leg wound complications developed in eight of 518 patients (1.5%) who received no-touch SV graft and underwent wound closure using the aforementioned strategies from 2017 to 2021 (unpublished data). All eight patients recovered after secondary intention healing.

## CONCLUSION

The rate of wound complication after not-touch SV harvest may be minimized by preoperative evaluation of lower limb vascular status, selection of an adequate vein, creation of a precise skin incision, careful harvesting of the vein, placement of a drain in the vein harvest site, and meticulous closure of the skin wound.

**Table:** 

Authors’ Roles & Responsibilities	
MSK	Substantial contributions to the conception or design of the work; final approval of the version to be published
SWH	Final approval of the version to be published
KBK	Agreement to be accountable for all aspects of the work in ensuring that questions related to the accuracy or integrity of any part of the work are appropriately investigated and resolved; final approval of the version to be published
